# Roles of microRNAs in abiotic stress response and characteristics regulation of plant

**DOI:** 10.3389/fpls.2022.919243

**Published:** 2022-08-26

**Authors:** Feiyan Zhang, Jiangwei Yang, Ning Zhang, Jiahe Wu, Huaijun Si

**Affiliations:** ^1^State Key Laboratory of Aridland Crop Science, Gansu Agricultural University, Lanzhou, China; ^2^College of Life Science and Technology, Gansu Agricultural University, Lanzhou, China; ^3^State Key Laboratory of Plant Genomics/Institute of Microbiology, Chinese Academy of Sciences, Beijing, China

**Keywords:** microRNA, target gene, abiotic stress, growing development, agronomic trait

## Abstract

MicroRNAs (miRNAs) are a class of non-coding endogenous small RNAs (long 20–24 nucleotides) that negatively regulate eukaryotes gene expression at post-transcriptional level *via* cleavage or/and translational inhibition of targeting mRNA. Based on the diverse roles of miRNA in regulating eukaryotes gene expression, research on the identification of miRNA target genes has been carried out, and a growing body of research has demonstrated that miRNAs act on target genes and are involved in various biological functions of plants. It has an important influence on plant growth and development, morphogenesis, and stress response. Recent case studies indicate that miRNA-mediated regulation pattern may improve agronomic properties and confer abiotic stress resistance of plants, so as to ensure sustainable agricultural production. In this regard, we focus on the recent updates on miRNAs and their targets involved in responding to abiotic stress including low temperature, high temperature, drought, soil salinity, and heavy metals, as well as plant-growing development. In particular, this review highlights the diverse functions of miRNAs on achieving the desirable agronomic traits in important crops. Herein, the main research strategies of miRNAs involved in abiotic stress resistance and crop traits improvement were summarized. Furthermore, the miRNA-related challenges and future perspectives of plants have been discussed. miRNA-based research lays the foundation for exploring miRNA regulatory mechanism, which aims to provide insights into a potential form of crop improvement and stress resistance breeding.

## Introduction

Plant microRNAs (miRNAs) are the important components in regulating gene expression or silencing by binding to complementary sequences with target messenger RNA(mRNA) to cause translational repression or transcript degradation (Griffiths-Jones et al., 2008; Voinnet, 2009; Ha and Kim, 2014). miRNAs control intricate regulatory networks and have been involved in a great diversity of biological processes in eukaryotic cells, which impact on plant development and response to biotic and abiotic stresses (Pegler et al., 2019; Secic et al., 2021). Both experimental and bioinformatics approaches have been adopted to identify miRNAs in plants. Early on, the direct cloning approach was widely used to discover miRNAs in plants (Llave et al., 2002; Reinhart et al., 2002). Then, as more information of highly conservation and stem-loop hairpin structure of miRNAs, bioinformatics approaches were developed to identify miRNAs in a new plant species from known miRNAs in other plant species (Jones-Rhoades and Bartel, 2004; Zhang et al., 2005). At present, based on high-throughput sequencing that has become more mature and affordable (Lu et al., 2006), small RNA high-throughput sequencing has been widely used to identify and functionally analyze miRNAs in plants (Sunkar et al., 2005; Fahlgren et al., 2007; Creighton et al., 2009). Owing to the rapid development of methods for identifying plant miRNAs, the increasing number of miRNAs has been discovered and identified in plants (Kozomara and Griffiths-Jones, 2011; Xu et al., 2016; Pegler et al., 2019). According to the records registered in miRBase (http://www.mirbase.org, release 22), 38,589 hairpin precursors and 48,860 mature microRNAs have been identified through experimental or computational approaches from 271 organisms, including more than 70 plants, such as *Arabidopsis thaliana* (326 precursors, 428 mature), *Oryza sativa* (604 precursors, 738 mature), *Zea mays* (174 precursors, 325 mature), *Triticum aestivum* (122 precursors, 125 mature), *Glycine max* (684 precursors, 756 mature), *Solanum tuberosum* (224 precursors, 343 mature), *Nicotiana tabacum* (162 precursors, 164 mature), *Solanum lycopersicum* (112 precursors, 147 mature), *Gossypium raimondii* (296 precursors, 296 mature), *Medicago truncatula* (672 precursors, 756 mature), *Populus trichocarpa* (352 precursors, 401 mature), *Sorghum bicolor* (205 precursors, 241 mature), *Brassica napus* (90 precursors, 92 mature), *Vitis vinifera* (163 precursors, 186 mature), and so on.

In the recent years, a crescendo of miRNA studies has demonstrated that miRNA-directed gene expression regulation is also essential for plants to adapt to the surrounding environmental stimuli (Sun X. et al., 2019). Suffering from abiotic stress, plants can strengthen tolerance to environment stimuli by regulating the expression of some miRNAs or producing some new miRNAs, so as to survive and grow better under the abiotic stress. Upregulating or downregulating the expression of corresponding miRNAs depends on different stresses, some miRNAs are also induced by several stresses in plants, such as high temperature, chilling, drought, high salinity, oxidization, and heavy metals (Jian et al., 2010). The studies based on stress-response miRNAs can provide important understanding into plant stress resistance breeding and gene expression, a powerful approach to unravel new insight into adaptive mechanism in plants.

As diverse as miRNAs are in response to individual or multiple environmental stimuli, so too are their responses to growth and development in plants. miRNAs have been characterized in regulation aspects of growth and development. Previous studies have shown that miRNAs involved in regulating plant flowering and floral organ development, leaf development, fruit development and ripening, root development, and embryogenesis (Lin and Lai, 2013; Gao et al., 2017; Correa et al., 2018; Plotnikova et al., 2019; Siddiqui et al., 2019; Yang et al., 2019). Recently, several important findings have proven that miRNAs and their targets are applied to regulate agronomic traits in crop plants, suggesting that miRNAs play an important role in the improvement of agricultural traits related to high yield and good quality, such as plant height, panicle branching, tillering, grain size, fruit quality, crop yield, male sterile, and so on (Wang S. et al., 2012; Zhang Y. C. et al., 2013; Yang et al., 2019).

In addition, miRNA could be a key regulator of plant secondary metabolism. The recent findings have shown the miRNAs give a pivotal role in regulating flavonoids, alkaloids, and nitrogen-containing compound biosynthetic pathway (Gou et al., 2011; Li et al., 2015; Davoodi Mastakani et al., 2018). No surprise, miRNA is also involved in the transduction of plant hormone signaling and regulation of metabolism pathway (Baek et al., 2013; Guo et al., 2018; Liu et al., 2019). miRNAs may regulate multiple target gene members in a gene family, and the certain target gene may also be involved in a variety of plant growth and development regulation processes.

Based on the functional diversification, this review gives a brief conclusion of miRNAs' roles in plants. miRNAs almost participate in all aspects of plant-growing development and primarily have influence on quality and yield of agronomic important plants, plant architecture, male or female fertility, organ development, and growing stages. In addition, abiotic stress conditions such as drought, high temperature, high salinity, and heavy metals can induce specific miRNA expression in plants, suggesting that miRNA can be used as the potential targets for agronomic trait improvement and stress tolerance formation in crop breeding.

## Abiotic stress-associated miRNAs in plants

Temperature, light, water, and mineral elements are the necessary environmental factors for plant growth and development. The abiotic stress response network mediated by miRNA is one of the important mechanisms of plant response to various abiotic stresses. Abiotic stresses are the main cause of crop losses worldwide. Most abiotic stresses, such as extreme temperature, drought, high salinity, and heavy metals, result in oxidative stress. Abiotic and oxidative stresses retard plant growth and diminish yield in model and crop plants (Kerchev et al., 2020). The miRNAs are implicated in abiotic stress response mechanisms with regard to oxidative stress and effects on DNA in different plant species (Pagano et al., 2021). As diverse as miRNAs are in response to growth and development in plants, so too are their responses to different environmental stimuli. miRNAs play a key role in responding to unfavorable conditions through regulating expression of related target genes in plants.

### miRNAs response to extreme temperature stress in plants

#### Low temperature

Low temperatures and frost compromise the plant survival and ultimately lead to growth retardation and yield loss (Das et al., 2021). Various phenotypic symptoms in response to chilling or freezing, including poor germination, stunted seedlings, yellowing of leaves, reduced leaf expansion, and wilting, and may lead to death of tissue (Ding et al., 2019). According to their targets, miRNAs respond to low-temperature stress through three tactics: the first is respond to abiotic stress directly; the second is indirectly responding to external stimuli by regulating transcription factors that relate to stress responses; and the third is that miRNAs can respond to multiple stresses and their target genes could code certain hydrolases or oxidoreductases (Yang et al., 2017).

**Figure 1:**
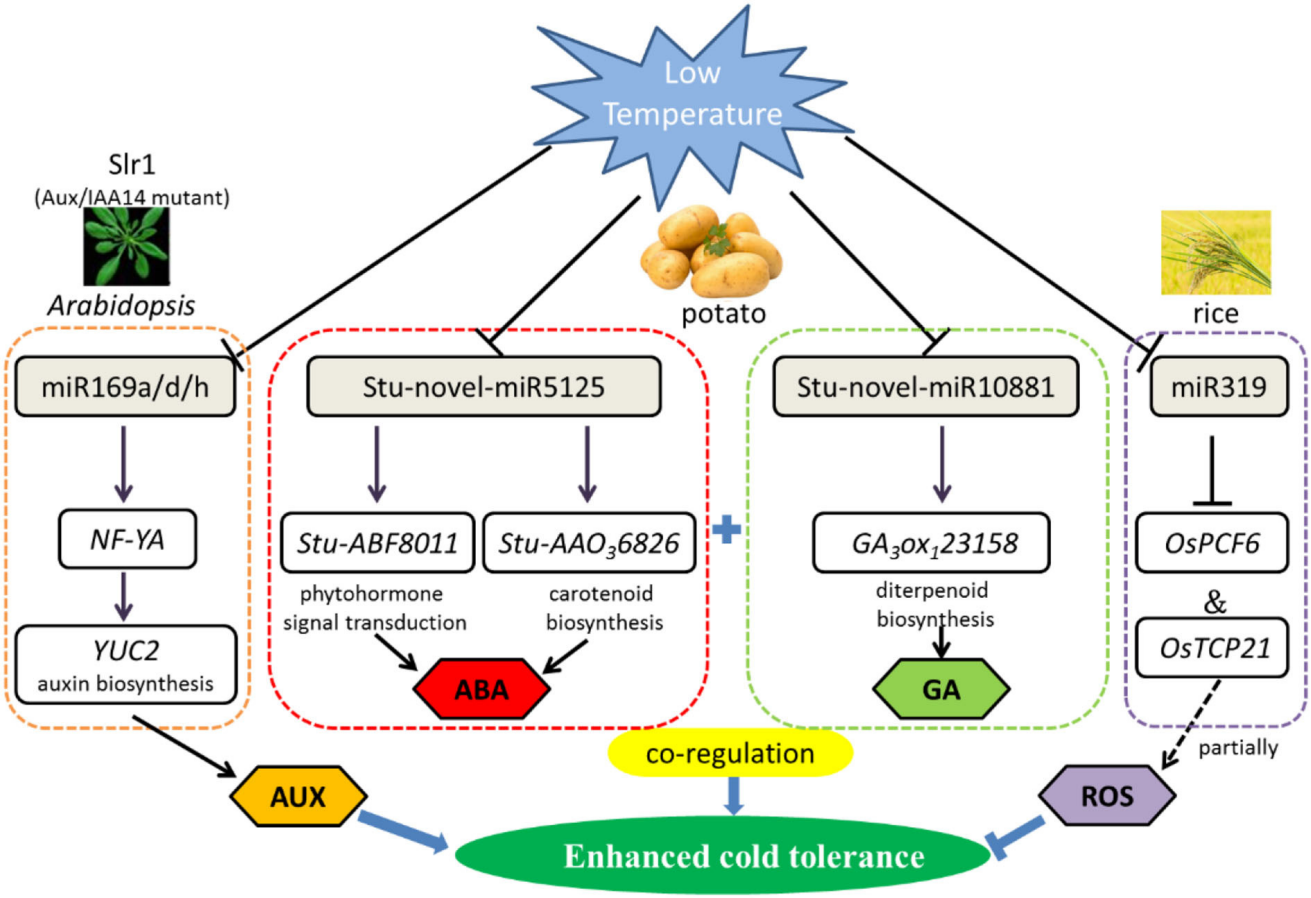
Regulatory network of the miRNAs involved in low-temperature stress primarily by phytohormone signal transduction and ROS scavenging mechanism in plants. Cold stress-responsive miRNAs in *Arabidopsis*, potato, and rice are shown in the chart. The arrows and hammer represent positive and negative regulation, respectively. AUX/IAA, Auxin/indole acetic acid; NF-YA, nuclear factor Y subunit A; YUC2, flavin-binding monooxygenase family protein 2; ABF, auxin F-box protein; AAO, abscisic-aldehyde oxidase; TCP, teosinte branched (tb1)-cycloidea (CYC)-proliferating cell factors1 and 2 (PCF1 and PCF2) transcription factor; ABA, abscisic acid; GA, gibberellin; ROS, reactive oxygen species.

Recent research in *Arabidopsis* roots reported that Aux/IAA14 regulates miRNA-mediated cold stress responding mechanism. Based on next-generation sequencing, 180 known and 71 novel cold-responsive miRNAs were revealed. Furthermore, comparative analysis of miRNA expression shows notable difference of 13 known and 7 novel miRNAs in slr1 (mutation in Aux/IAA14) and wild types. Interestingly, compared with wild type, miR169 was downregulated in slr1 after 12-h cold treatment at 4°C, particularly in the miR169a, miR169d, and miR169h. As miR169 target gene, NF-YA regulates the auxin biosynthesis gene YUC2 (Figure 1). Therefore, miR169 plays a crucial role in cold tolerance by regulating auxin (Aslam et al., 2020).

There is an example for miRNA and their predicted targets involved in regulation of maize growth and development under low-temperature stress (Aydinoglu, 2020). After chilling stress treatment (4°C), there were a large number of miRNAs with altered expression in different developmental zones in maize leaf, suggesting that miRNAs play a vital role in modulating the leaf developmental adjustments required for adaptation to plant against low temperature in maize. The expression of miR160 and miR319 was significantly decreased in the mature zone. However, miR408a and miR528 were upregulated in the meristem and elongation zones. Compared with the control, the expression levels of miRNAs have changed under chilling stress of the maize.

Some conserved miRNAs were first discovered to respond to cold stress in potato seedlings, for instance, stu-miR530, stu-miR167b, and stu-miR156d. Intriguingly, the number of miRNAs declines as the temperature decreased (2, 0, and −2°C), both of known and novel miRNAs. Gene Ontology (GO) and Kyoto Encyclopedia of Genes and Genomes (KEGG) pathway enrichment analysis show that miRNAs respond to cold stress by regulating circadian rhythm, carotenoid biosynthesis, and plant hormone signal transduction metabolic pathways in potato (Figure 1). With low-temperature treatment, qRT-PCR analysis clearly showed that StuABF8011 and StuAAO36826 were significantly negatively targeted by stu-novel-miR5125, which regulated abscisic acid metabolic pathways. Gibberellin 3beta-dioxygenase (GA3ox1) is a key enzyme of gibberellin synthesis, and in the same way, GA3ox123158 is also negatively regulated by stu-novel-miR10881. The results pointed out that miRNAs display a significant superposition or coordination role in resisting cold stress through regulating gibberellin and abscisic acid signal transduction network (Yan et al., 2021).

Except for participating in miR168-regulated immunity, a new function of miR1320 responding to abiotic stress has been identified recently. Overexpression of miR1320 resulted in increased cold tolerance in rice. AP2/ERF TF *OsERF096*, as a target of miR1320, co-regulate cold tolerance by repressing the JA-mediated cold signaling pathway (Sun et al., 2022). Similarly, overexpression of Osa-miR156, Osa-miR319, and Osa-miR528 also can improve cold resistance in rice (Huo et al., 2022). In addition, miR319 positively regulates cold tolerance by targeting OsPCF6 and OsTCP21 in rice (Figure 1), and the downregulation of these two transcription factors resulted in enhanced tolerance to cold stress (Wang et al., 2014). Besides, the 105 potential miRNA target genes were predicted by degradome sequencing of alfalfa under cold (4°C) and freezing (8°C) for 3 h, among 28 highly enriched transcription factors from 13 families were identified, such as SBP, ARF, NAC, AP2/ERF, CCAAT, and GRF, which could be cleaved by miR156, miR160, miR164, miR5205, miR169, and miR396, respectively, all of them play a decisive role in resisting abiotic stress (Shu et al., 2016). These findings represent a foundation for exploring the molecular mechanisms of miRNAs enhanced low-temperature tolerance in plants.

#### High temperature

Due to global warming and climatic changes, heat stress gradually becomes one of the restricted factors of growth and development in plants. The sustainability of agricultural production is being threatened by high-temperature stress. To alleviate the adverse effects, mainly manifest in photosynthesis, respiration, nitrogen metabolism, protein turnover, nucleic acid metabolism, and lipid metabolism, heat-responsive miRNA has been studied extensively (Grover et al., 2013; Zuo et al., 2021; Begum, 2022).

It is reported that the miRNA-mediated post-transcriptional regulation could respond to high temperature in maize. Prominent phenotypic responses of maize leaves at 40/25°C (day/night), at the same time, ROS produced and accumulated, as well as ROS-induced membrane lipid peroxidation, showed significant increases. This study revealed 61 known and 42 novel miRNAs displaying significant differential expression. Among the rest, miR169 family had the highest number in differentially expressed miRNAs, followed by the miR159, miR156, and miR160 families, downregulating under high temperature. Several important regulatory miRNA-target modules involved in heat stress have been found, such as miR169-SBP, miR159-MYB, miR156-SBP/SPL, miR172-AP2/ERF, miR164-NAC, miR166-HD zip, miR396-GRF, and miR5381-SAC2. More interestingly, the regulation pattern based on the expression of miRNAs is plant-specific under heat stress (Zhang et al., 2019). The most probable reason is that different plant species have different treatment conditions. It is difficult to generalize a rule of regulation of miRNAs under heat stress conditions. miR156, as the first miRNA identified in plants and plays a positive role resist high temperature, is highly induced. miR156 target SPL transcription factor gene is downregulated at transcriptional level after heat stress (Stief et al., 2014). Another study indicated that the miR156-SPL module might regulate the heat stress in banana, and SPL gene family co-targeted by miR535 and miR156. Abundant miRNAs all showed downregulate expression trend in response to high-temperature stress in bananas. Overall, high-temperature stress tended to inhibit miRNA expression (Zhu et al., 2019). In addition, different miRNAs resist high temperatures in several pivotal stages of anther development in cotton. Among the rest, miR2949, miR167, and miR160 at the sporogenous cell proliferation stage; miR156 and miR172 at the meiotic phase stage; miR156 at the microspore release period; and miR393 and miR3476 at the pollen maturity stage. These miRNAs served as the main regulators responding to stress in the heat-tolerant and the heat-sensitive cotton lines (Chen J. et al., 2020).

The study on miRNAs that participate in tomato stamen and pistil development under high temperatures (35°C) discovered that the miR167, miR396, and miR482 families were significantly expressed in the stamen, whereas the miR159, miR166, and miR482 families were expressed in the pistil. These miRNA families show conserved functions in response to heat stress. It is worth mentioning that no miRNAs were predominantly upregulated under heat stress in either the tomato stamen or pistil, indicating that long-term exposure to heat stress could suppress the expression of miRNAs. Furthermore, the following results were obtained through GO and KEGG pathway prediction, qRT-PCR, and RLM-5′RACE validation, miR393-SlTIR1 and miR160-SlARF10/16 were participated in the regulation of auxin signaling pathway, and miR398b-SlCSD1 and miR397-LACs were tightly correlated with ROS-mediated cell expansion and cell division. At the same time, miR156-SPL emerged as a pivotal regulator to induce multiple aspects in plants, both of which were activated by heat stress treatment (Pan et al., 2017). Last but not least, the heat-tolerant wild tomato at the moderately elevated temperature (33°C) showed opposite expression, and miR156 and miR396 were upregulated expressions. Inversely, miR168 acted out downregulated expression. Nevertheless, at the acutely elevated temperature (40°C), miR319 and miR398 were downregulated expressions. The above results further illustrated that extreme high temperature is likely to restrain the expression of miRNAs (Zhou et al., 2016).

In addition, artificial miR160 mimics an inhibitor of miR160, which showed worse adaptation to heat stress. On the contrary, overexpressing miR160 significantly enhanced abilities of thermotolerance in transgenic *Arabidopsis*. Target genes of miR160, including ARF10, ARF16, and ARF17, were repressed by miRNAs, which regulate the expression of the heat shock proteins to survive under heat stress (Lin et al., 2018). Overexpression of miR156 also enhances alfalfa tolerance to heat stress (Arshad et al., 2020). Additionally, miR156, miR159, miR168, miR171, and miR1885 were found in flowering Chinese cabbage after 38°C heat stress treatment (Ahmed et al., 2019). miR156, Tae-miR818, miR169, miR528, and miR398 were downregulated in response to heat stress in wheat (Ragupathy et al., 2016). In addition, the role of heat-responsive miRNAs, miR528, and miR9662 in regulating the antioxidant activity and stress-responsive mitochondrial proteins was revealed in wheat (Ravichandran et al., 2019). The terminal heat stress critically affects wheat quality and yield. miRNA-based SSR markers, as the key players in improving terminal heat-tolerant wheat, have been validated on the terminal heat-tolerant and heat-susceptible genotypes in the early stages of wheat development. Notably, two very promising diagnostic markers, miR159c and miR165b, showed specific alleles and discriminated terminal heat-tolerant genotype, which are involved either directly or indirectly in providing tolerance to heat stress (Sihag et al., 2021). Also, AGO1 has been reported to be targeted by miR168 in *Populus* during heat stress responses. The repressed expression of miR168 result in overexpression of AGO1 under heat stress, suggesting that the miRNA-mediated regulation system is more active under heat stress (Chen et al., 2012).

**Figure 2:**
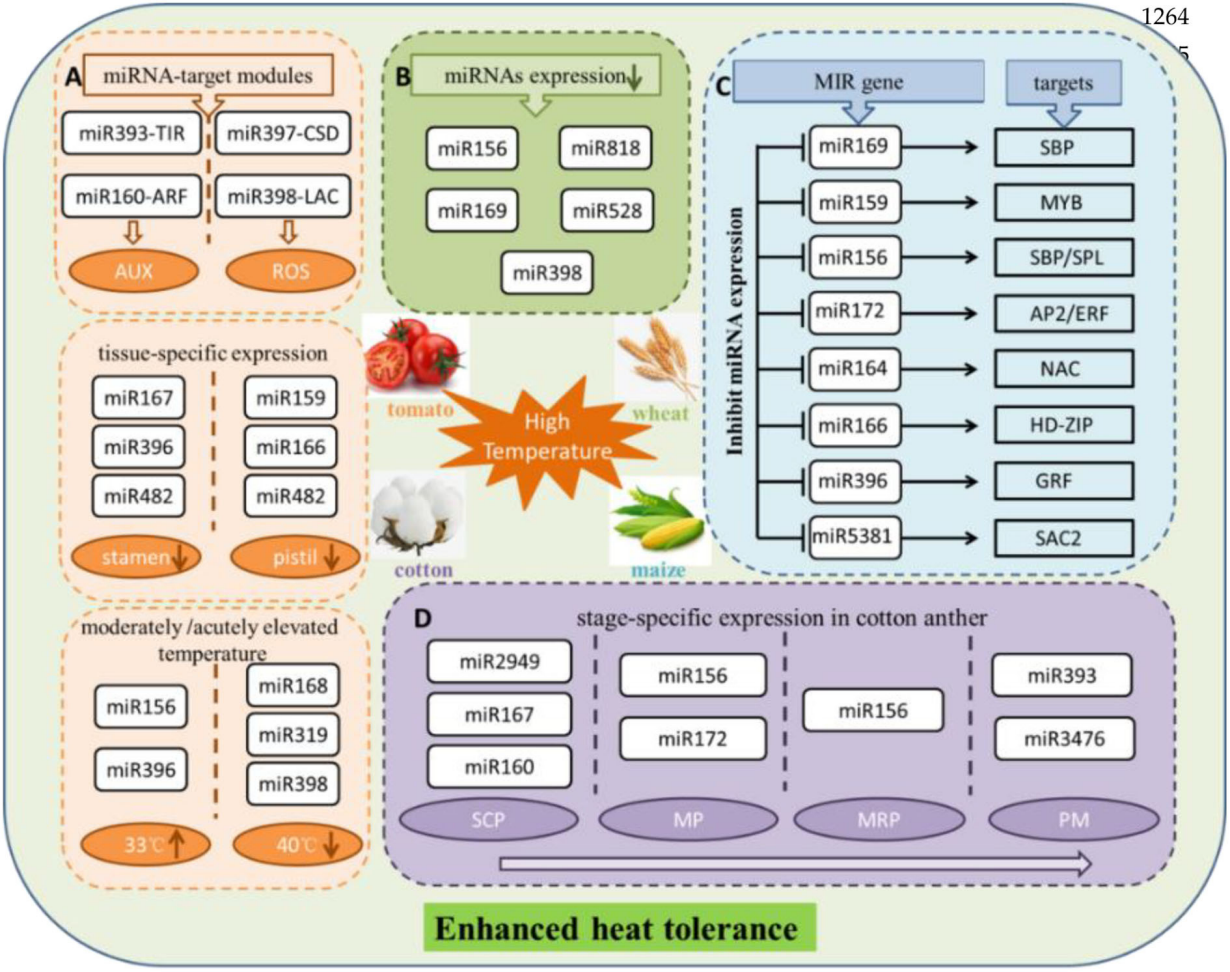
Schematic diagram exhibited that multiple miRNAs in response to high-temperature stress in several plants. **(A)** MiRNAs display tissue-specific expression under heat stress in tomato and through regulating different metabolic pathways to resist high temperature. Also, miRNAs expression is affected by moderately or acutely elevated temperature. **(B)** Certain miRNAs are showed downregulated expression suffered heat treatment in wheat. **(C)** Effect on expression of miRNAs and their corresponding targets under heat surrounding conditions in maize. **(D)** MiRNAs show specific expression in critical developmental period of cotton anther to adapt to high-temperature stress. SCP, sporogenous cell proliferation stage; MP, meiotic phase stage; MRP, microspore release period; PM, pollen maturity stage. miRNAs upregulated expression and downregulated expression are represented by upward and downward arrows in the circle, respectively. AUX, auxin; ROS, reactive oxygen species; MYB, MYB proto-oncogene transcription factor; SBP/SPL, SQUAMOSA promoter-binding proteins; AP2/ERF, ethylene response factor; NAC, NAM, ATAF and CUC (NAC) transcription factors; HD-ZIP, zipper motif (LZ) immediately downstream of the home domain; GRF, growth-regulating factor1; SAC2, adenylate cyclase.

In conclusion, previous reports showed that species-specific miRNAs are usually expressed at a lower level (Fahlgren et al., 2007). With the increase of research, a large number of plentiful conserved and species-specific novel miRNAs have been identified in many important crops, and these species-specific miRNAs obtained stable and specialized functions (Pan et al., 2017; Chen J. et al., 2020). miR824, miR858, miR856, and miR857 are the *Arabidopsis*-specific miRNAs, the target gene of miR824 is AGL16 (At3g57230, MADSbox), the target gene of miR858 is MYB12 (At2g47460, R2R3-MYB family), and target gene of miR856 is an unrelated transcript encoding the efflux protein ZAT1, but the functions of these targets are not known. Also, miR857 was validated to target a laccase family gene LAC7 (At3g09220) transcript with predicted multicopper oxidase function. miRNAs are involved in response and mitigation of the detrimental effects related to high-temperature stress by regulating the abundance of transcription factors and proteins associated with the metabolism of reactive oxygen species (ROS), as well as in several mitogen-activated protein kinases (MAPK) signaling pathways (Ahmed et al., 2019; Zhang et al., 2019). miRNAs were demonstrated the positive or negative regulation in heat stress adaptation and heat stress tolerance (Figure 2).

### miRNAs response to drought stress in plants

Drought is one of the most adverse abiotic factors in constraining growth and development in plants, which has significantly impacted crop productivity and posing a threat to world food security (Fahlgren et al., 2007). Stress can result in a series of physiological and biochemical reactions in plants (Zhu, 2016). To enhance the performance of plant and better understanding the molecular mechanism under drought, the research of miRNA is highly desired. Increasing evidence suggests that drought-responsive miRNA has been investigated in several dicotyledonous and monocotyledonous species, for instance, *Arabidopsis* (Ni et al., 2012; Li et al., 2016; Yang et al., 2019; Yu et al., 2019), maize (Das and Mondal, 2021), alfalfa (Arshad et al., 2018), cereals (Geng et al., 2021; Singroha et al., 2021), cardamom (Anjali et al., 2018), barley (Ferdous et al., 2017), soybean (Zhou et al., 2020), and potato (Yang et al., 2014).

Refer to drought stress, the phytohormone abscisic acid (ABA) is involved in initiating an adaptive response to withstand water depletion through regulating stomatal conductance. ABA also coordinates with other plant hormones to resist drought stress, such as salicylic acid (SA), auxin (AUX), gibberellins (GA), ethylene (ET), brassinosteroid (BR), jasmonic acid (JA), and so on. ABA-mediated and non-ABA-mediated drought resistance pathways are remarkably regulated by miRNAs. Most miRNA target genes responding to drought stress are the transcription factors, such as MYB, NF-Y, NAC, and HD-ZIP. For instance, miR159-MYB module and miR169-NFYA module are involved in an ABA-dependent pathway. However, several miRNA-target modules (miR156–SPL; miR393–TIR1; miR160–ARF10, ARF16, ARF17; miR167–ARF6 and ARF8; and miR390–ARF2, ARF3, ARF4) participate in an ABA-independent pathway (Singroha et al., 2021).

To date, *Arabidopsis* as a model species has been widely studied on the reaction and expression of miRNAs and their target genes under drought stress. In *Arabidopsis*, research on miR160 and miR165/166 has an effect upon leaf growth and development and drought tolerance through the technology of short tandem target mimic (STTM), and the drought tolerance of the hybrid strain STTM160 × STTM165/166 was significantly stronger than the single mutant STTM160, but weaker than the double-mutant STTM165/166. This study manifested that miR165/166-mediated transcription factor HD-ZIP IIIs regulation can conquer water limitation by ABA signaling pathway (Yang et al., 2019). Under the drought treatment, overexpression of soybean miR169c that exerts a negative regulatory role by inhibiting the expression of its target genes AtNFYA1 and AtNFYA5 transcription factors, which resulted in gma-miR169c-overexpressing *Arabidopsis* increased drought stress sensitivity, led to the leaves water deficit and reduced plant survival rate (Yu et al., 2019). The same thing is that overexpressing gma-miR398c in *Arabidopsis* and soybean resulted in decreased ability of drought resistance through negatively regulating multiple peroxisome-related genes (GmCCS, GmCSD1a/b, and GmCSD2a/b/c) (Zhou et al., 2020). However, on the contrary, overexpression of gma-miR394a confers tolerance to drought stress in transgenic *Arabidopsis* (Ni et al., 2012). Similarly, that overexpression of gma-miR172c in *Arabidopsis* exhibits increased tolerance to drought and high salt (Li et al., 2016).

Accumulating evidence has proven that miRNAs are involved in imparting drought stress in economically important crops. Recently, the research about prediction of miRNA targets by degradome analysis revealed the genotype-specific miRNA regulation under drought stress in barely. Differential expression of miR169 and miR1432 has been observed between sensitive and tolerant genotypes. Compared to the well-watered barely, higher expression of miR169b was detected in the drought-stressed barely in mesophyll tissues (Ferdous et al., 2017). Besides, 17 miRNAs belonging to 11 families were found to respond to water deprivation in monocot plant cardamom. Water deficit reduced miRNA-target module (miR159- MYB, miR396-GRF, miR535-SPL, miR166b-HD-ZIP III, and miR167-ARF) expression in cardamom. Interestingly, miR172 was significantly downregulated. Similarly, the expression of miR156l was downregulated in cardamom. However, miR169f, miR156d, and miR159b were upregulated. To sum up, in response to drought stress, plants manifested different regulations in the same species to adapt to hydronic conditions. It is worth mentioning that as miR396d and miR396e were observed only in cardamom, miR528 as a monocot-specific miRNAs also was detected in rice, sorghum, and maize (Anjali et al., 2018).

Furthermore, overexpression of miR156 contributed to combat drought in alfalfa. Major transcription factors affected by drought stress in miR156OE transgenic lines belong to transcription factors bHLH, HD-ZIP, TCP, C2H2, and WRKY families. Further, overexpressing WD40-2 strains showed drought sensitive, whereas silenced WD40-2 strains exhibited drought tolerance. Overexpressing and RNAi plants exhibited opposite effects. Hence, research suggests WD40-2 as miR156 target gene, which is a negative regulator in response to drought tolerance in alfalfa (Arshad et al., 2018). Moreover, stu-miR159 family members negatively regulated the GAMyb-like genes (StGAMyb-like1, StGAMyb-like2.1, and StGAMyb-like2.2) under drought stress in potato. These three genes belong to conserved plant R2R3 MYB domain transcription factors. Therefore, suppression of stu-miR159s could cause a better adaptability to drought stress (Yang et al., 2014). In addition, the overexpression of Sit-miR394 in transgenic *Arabidopsis* increased tolerance under drought stress. MiR394 is also regulated by multiple phytohormones such as ABA, ETH, JA, and SA. Conserved Sit-miR394 target gene SiFBP6 positively regulates drought resistance in foxtail millet (Geng et al., 2021).

In maize, several reported miRNAs (such as miR159, miR160, miR167, miR389a, miR393, miR397b, miR402, and miR474) were revealed to fine-tune their target gene expression to resist drought stress, and several regulons such as MYC/MYB, NAC, DREB/CBF, ABRE, ZF-HD, and PDH are involved in drought stress. miR398a-MYB101/MYB33 and miR164-NAC were downregulated, whereas miR393-MYB101/MYB33 and miR402-MYB101/MYB33 were upregulated. Both of them were related to ABA-mediated response pathway. Upregulation of miR160-ARF10 involved in auxin response pathway and down-regulation of miR528-POD involved in ROS detoxification pathway, respectively. It is worth mentioning that miR167-PLD was downregulated in maize and rice, but upregulated in *Arabidopsis* by responding to ABA-mediated signal pathway in drought resistance (Das and Mondal, 2021). Also, maize miR474 was upregulated and the target gene of PDH was downregulated under drought stress. It is used to inhibit proline accumulation to improve the osmotic protective in response to drought stress (Wei et al., 2009). In *Medicago truncatula*, expression of miR398a/b and miR408 was upregulated under drought stress in both roots and shoots, the upregulated miR398a/b and miR408 led to the downregulation of their target transcripts, copper superoxide dismutase (CSD1/2), mitochondrial cytochrome coxidase, and plastocyanin, which establish a direct link in the adaptation of *M. truncatula* to drought and copper homeostasis (Trindade et al., 2010).

As one of the most important crop plants, wheat provides calories for a large part of the population. Drought-responsive miRNAs have been investigated in wheat (Singroha et al., 2021). The mutation led to the loss of tae-miR9657b-5p targeting, and lost the binding of tae-miR9657b-5p, which caused increased expression of tae-miR9657b-5p under the drought conditions, indicating that post-transcriptional RNA modification might mediate miRNA to response the drought stress (Pan et al., 2022). Moreover, according to the first durum wheat degradome report, some new miRNA-target regulatory pairs were discovered. The reduced abundance of osa-miR160f-5p and ata-miR396c-5p resulted in increased expression of NDH and PGR5 in durum wheat. The activity of NDH and PGR5 increased significantly under the drought stress. These two miRNA-target modules confer enhanced drought tolerance by protecting the photosynthetic apparatus and resulting in the lower levels of ROS accumulation. Also, the ata-miR528-5p-target module could contribute to remain redox homeostasis (Liu et al., 2020). These modules could be the promising candidates for improving drought resistance in durum wheat.

**Figure 3:**
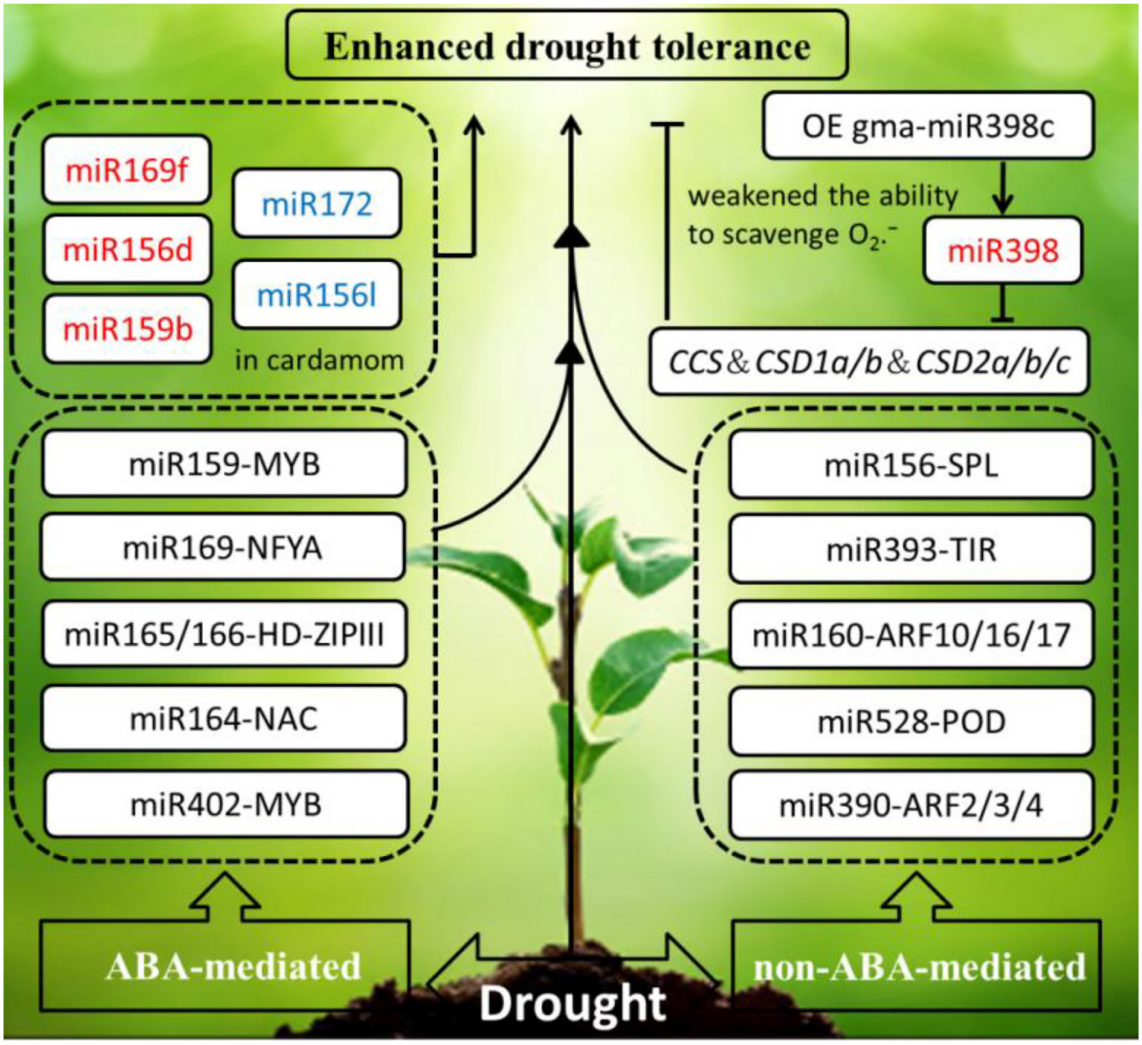
Schematic diagram of the drought-responsive miRNAs in plants are primarily by phytohormone ABA-mediated and non-ABA-mediated pathways initiating an adaptive response to enhance drought tolerance. The miRNAs in red and blue font were upregulated and downregulated under drought stress, respectively. The arrows and hammer represent positive and negative regulation, respectively. OE, overexpression; MYB, MYB proto-oncogene transcription factor; NF-YA, nuclear factor Y subunit A; HD-ZIPIII, zipper motif (LZ) immediately downstream of the home domain; NAC, NAM, ATAF, and CUC (NAC) transcription factors; CCS, copper chaperone for superoxide dismutase; CSD, copper-zinc superoxide dismutase 1and 2; SPL/SBP, SQUAMOSA promoter-binding proteins; TIR, transport inhibitor response; ARF, auxin response factors; POD, peroxidase; ABA, abscisic acid.

In summary, the drought-responsive miRNAs in plants primarily by phytohormone ABA-mediated and non-ABA-mediated pathways are summarized in the schematic diagram (Figure 3), the miRNA response to drought stress is highly intricate, and their altered expression is influenced by treatment methods, tissue, genotype, and developmental stage (Ferdous et al., 2015).

### miRNAs response to salt stress in plants

Salinization is a worldwide environmental issue. Salinity exerts detrimental constraints on global plant growth, development, and crop yield, posing a serious challenge for plant breeding (Wani et al., 2020). Salt stress affects plants in a complex way *via* 3-fold impacts, viz. hyperosmotic stress, ion toxicities, and imbalances, as well as oxidative stress (Kumar et al., 2018). When the salt concentration exceeds its normal level, water potential declines in the soil leading to the reduced absorption of water by plant roots. The saline environment has severely affected and will continue to affect the growth of plants (Ibrahimova et al., 2021). Based on this, it is very necessary to enhance salt tolerance in crop plants. Numerous evidence has shown that miRNAs as critical post-transcriptional regulators in plants are involved in salt tolerance and have an important effect on salt stress response (Sun X. et al., 2019).

Salt-related studies mainly focused on the *Arabidopsis*, miR397 from the *Setaria viridis*, a kind of C4 monocotyledonous grass model species. Overexpressed SvMIR397 or AtMIR397 obtained the transformed strains, under 150 mM sodium chloride (NaCl) treatment in 7 days, could repressed the expression of target gene LACCASE (LAC), especially caused a significant decrease in LAC2, LAC4, and LAC17, which led to lignin content reduced and salt stress sensitivity enhanced in *Arabidopsis* (Nguyen et al., 2020). Additionally, miR393 could regulate salinity stress through scaffold protein RACK1A-mediated ABA signaling pathways in *Arabidopsis* seedlings under exogenous 100 mM NaCl salt treatment. Due to TIR1/AFB2 is targeted by the miR393, RACK1 is a negative regulator of phytohormone ABA. MiR393 is implicated in salt acclimation through an antagonistic response between the ABA-mediated salt stress and hormone auxin (Denver and Ullah, 2019). Interestingly, Ding et al. (2009) find 18 miRNAs expressed in salt-tolerant maize lines, whereas 25 miRNAs in salt-sensitive lines show a delayed regulation pattern (Ding et al., 2009; Kumar et al., 2018).

As reported in the last few years, plants protect themselves from the adverse effects of salts by regulating miRNA, except for model plant *Arabidopsis*, in a variety of other plant species viz. alfalfa (Arshad et al., 2017), cotton (Wang et al., 2019), apple (Ma et al., 2021), switchgrass (Liu et al., 2019), Jerusalem artichoke (Wen et al., 2020), tamarisk trees (Ye et al., 2020), cereal crops (Cheng et al., 2021), etc. involved in the salt tolerance. Overexpression of miR156 through downregulated SPL transcription factor family genes could improve biomass accumulation and forage quality. On the other hand, miR156 modulates ion homeostasis under salinity stress in alfalfa. Increased miR156 expression levels tend to reduce the uptake of Na+ under severe salt stress (Arshad et al., 2017). In contrast, miR156a overexpression decreases salt tolerance. However, MdSPL13 overexpression improves salt tolerance in apples treated with 150 mM NaCl. Analyzing the relationship between miR156 and MdSPL13 by RLM-5'RACE revealed that the miR156-SPL module regulates salt stress tolerance by activating MdWRKY100 expression. Furthermore, MdSPL13 binds and activates the MdWRKY100 promoter which was verified by electrophoretic mobility shift assay (EMSA) and chromatin immunoprecipitation (ChIP-qPCR). Therefore, the expression of miRNA and/or their targets can be exhibited the salt tolerance (Ma et al., 2021).

In addition, miR319 has been defined as an important regulator in abiotic stress tolerance in both C3 and C4 plants. By comparing OE-miR319 (overexpression of Osa-miR319b) and MIM319 (inhibited miR319) switchgrass (*Panicum virgatum* L.), both of them were treated with 300 mM NaCl, which demonstrated that miR319 positively regulated ethylene synthesis and salinity resistance. As expected, repression of PvTCPs, notably PvPCF5, the miR319 target gene, also led to promote ethylene accumulation and salt stress tolerance (Liu et al., 2019).

The concentration of salt induced different expressions of miRNAs. The analysis of salt stress-responsive miR390 in Jerusalem artichoke (*Helianthus tuberosus* L.), when treated with high concentration of NaCl (300 mM), inhibited the expression of miR390, attenuated cleavage of TAS3 protein (specifically paired with miR390), produced low levels of tasiARFs (trans-acting small interfering RNAs influencing ARF), and subsequently reduced the cleavage of ARF3 and ARF4 genes. It is worth mentioning that, in contrast, 100 mM NaCl treatment reduced miR390 expression. Thereby, miR390-TAS3-ARF pathway has an instrumental function in response to salt stress (Wen et al., 2020). Another research suggested that a potential miR167 target gene, TcARF6, was significantly downregulated specifically in the *Tamarix chinensis* roots after 340 mM NaCl solution immersion (Ye et al., 2020).

Plants through the antioxidant defense system resulted in increased salt tolerance. miRNAs could modulate salt-triggered ROS scavenging. Proven studies have shown that miR172 is a positive regulator of salt tolerance. IDS1 as one of the downstream target genes of miR172 binds to the promoters of ROS-scavenging genes for transcriptional repression. Thus, miR172-IDS1 displays obvious regulatory relationships in maintaining ROS homeostasis during salt stress in cereal crops such as rice and wheat (Cheng et al., 2021). Furthermore, studies have found that miR414c affects salt tolerance in cotton by regulating reactive oxygen species (ROS) metabolic. Overexpressing miR414c depressed the expression of GhFSD1 (a cotton iron superoxide dismutase gene), due to FSD1 represents the first line of defense against stress-induced ROS, an excessive hypersensitive phenotype to salinity stress is noticeable (Wang et al., 2019).

**Figure 4:**
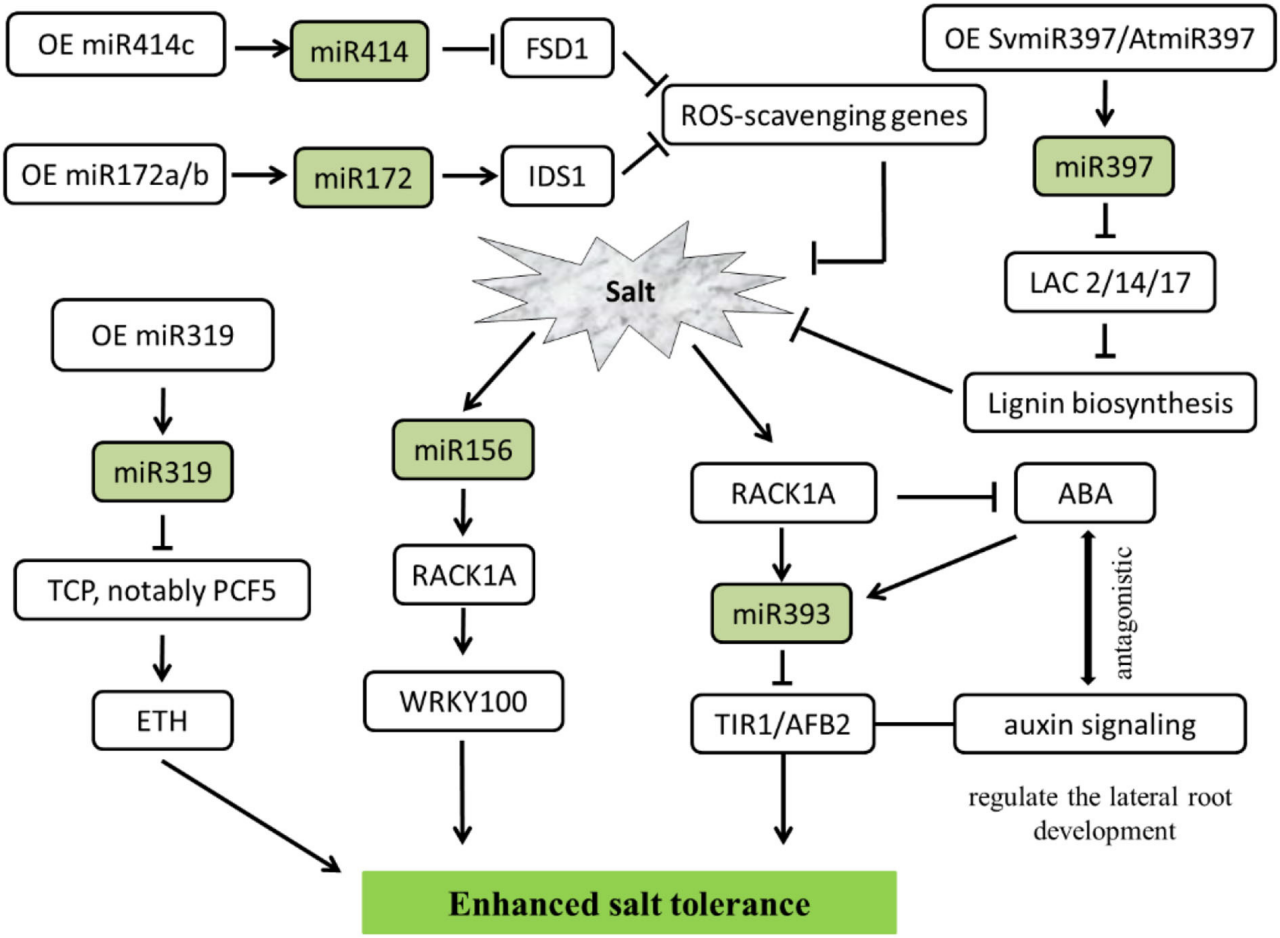
MiRNA-mediated regulating network responses salt stress in plants. The arrows and hammer represent positive and negative regulation, respectively. OE, overexpression; ROS, reactive oxygen species; ETH, ethylene; ABA, abscisic acid; FSD1, iron superoxide dismutase 1; IDS1, INDETERMINATE SPIKELET 1; LAC, LACCASE enzymes; TCP/PCF, teosinte branched (tb1)-cycloidea (CYC)-proliferating cell factors1 and 2 (PCF1 and PCF2) transcription factor; RACK1A, receptor for activated C kinase 1; WRKY, WRKY transcription factors; TIR, transport inhibitor response; AFB, auxin F-box protein.

At the same time, salt-inducible miRNA is a key upstream molecule in regulating the salinity stress signal, which could help plants grow normally in high salt environments. The overdose accumulation of ROS in plants was reduced, thereby conferring salt tolerance to plants. In brief, the transgenic strategy paved a way for overexpressing or repressing some important miRNA target genes viz. ARF, IDS, AFB, LAC, SPL, TCP, etc. conferred salt stress tolerance (Figure 4).

### miRNAs response to heavy metal stress in plants

Heavy metal (HM) stress is a major yield-limiting factor for plants (Ding et al., 2020). Some HMs such as manganese (Mn), copper (Cu), boron (B), zinc (Zn), and iron (Fe), at lower concentration, play a positive role, which is an essential component of plant cell. On the contrary, at higher concentration, these HMs impose toxic reaction in the plants, which have a strong negative impact on plant growth and development. Other metals, such as cadmium (Cd), aluminum (Al), arsenic (As), plumbum (Pd), and mercury (Hg), are potentially highly toxic to plants (Gupta et al., 2014). Previous studies have shown that miRNAs, as one of the plant survival strategies, are the prospective target to regulate heavy metals tolerance in plants (Noman and Aqeel, 2017).

MicroRNAs involved in the response to superfluous heavy metal elements Cd and Cu are identified by the use of a spl7 knockout mutant in *Arabidopsis thaliana*. Transcription factor SPL7 is the key regulator of Cu homeostasis, because of the Cu homeostasis affected by Cd, and thus, Cd-induced Cu deficiency caused that miRNAs, such as miR857, miR397a, and miR398b/c, were oppositely affected under Cu and Cd exposures (Gielen et al., 2016). In *Brassica napus*, 84 miRNAs and 802 target genes were identified using small RNA high-throughput sequencing and degradome sequencing. Among them, the gene that encodes NRAMP metal transporter was identified as the target gene of miR167 (Ding et al., 2018). BnNRAMP1b was further confirmed to be targeted by miR167 in *B. napus* (Meng et al., 2017). In addition, a set of miRNAs response to cadmium stress were identified from *B. napus* (Huang et al., 2010), and miR395 was reported to involved in detoxification of cadmium in *B. napus* (Zhang L. W. et al., 2013). On the other hand, the same metal stress induces contrastive responses of miRNAs in different species. miR167 was upregulated in *Arabidopsis* but suppressed in maize upon salt stress (Khraiwesh et al., 2012).

Besides, another research discovered a pivotal role of miR166 in Cd accumulation and tolerance by the regulation of its target gene OsHB4 in rice (Ding et al., 2018). Overexpression of miR156 improved antioxidative capacity and decreased Cd accumulation in *Arabidopsis* shoot (Zhang et al., 2020). In addition, miR319 and miR171 play a crucial role in adaptation to long-term B toxicity by targeting MYB transcription factor gene and SCARECROW-like protein gene, respectively, and both of them are responsible for root growth and development in citrus (Huang et al., 2019).

## Growth and development-associated miRNAs in plants

**Figure 5:**
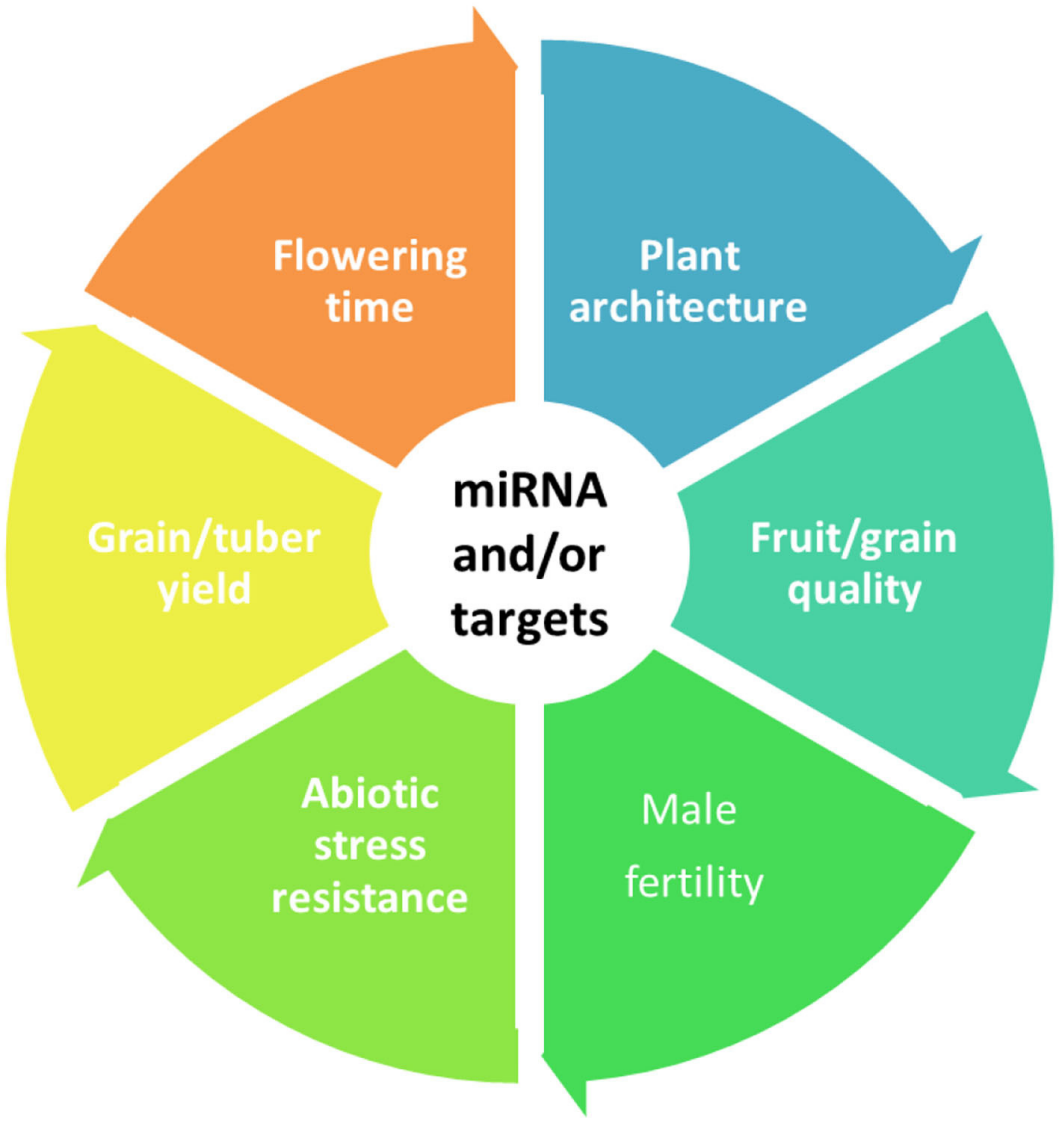
MiRNAs and/or their target genes regulate multiple agronomic traits of plants. Primarily for quality and yield of agronomic important plant, ideal plant architecture, abiotic stress resistance, male sterility, and flowering time.

With the global environment and climate changing in the world, focus on traits with the greatest potential to increase yield is necessary. A number of genes related to plant agronomic traits have been identified, and quite a number of these genes have been proved to be regulated by miRNAs. Based on functional diversification, miRNAs almost participate in all aspects of plant traits, primarily for quality and yield of agronomic important plant, ideal plant architecture, abiotic stress resistance, male sterility, and flowering time (Figure 5; Table 1).

**Table 1:** A list of miRNAs and their targets are involved in the regulation of growth and development in various plants.

**miRNAs**	**Species**	**Expression characteristic**	**Targets**	**Target functions**	**References**
miR156	*Arabidopsis*	Up	SPL	Leaf number and morphology, shoot architecture	Kim et al., 2017; He et al., 2018
	Soybean	Up	SPL	Numbers of long branches, nodes, and pods	Sun Z. et al., 2019
	Maize	Up	SPL	Juvenile stage development	Chuck et al., 2007
	*Arabidopsis*	Up	SPL/SBP	Heteroblastic development	Nguyen et al., 2017
	Tomato	Up	SPL	Ovary and fruit development	Ferreira e Silva et al., 2014
	Alfalfa	Up	SPL	Root length and biomass	Aung et al., 2015
	Potato	Up	SPL	Plant architecture and tuber yield	Bhogale et al., 2014
	Rice	–	SPL14	Plant height, tillering, size quality and shape of rice grains; root development	Jiao et al., 2010
	Rice	–	IPA1	Enhance seed dormancy, modify shoot architecture and increase grain size	Miao et al., 2019
	*Arabidopsis*	Down	AGAMOUS-like protein	Floral development	Serivichyaswat et al., 2015
miR160	*Dimocarpus longan*	–	ARF10/16/17	Embryo development, hormone signaling transduction	Lin et al., 2015
	Soybean	Up	ARF	Symbiotic nodule development	Turner et al., 2013
	*Arabidopsis*	–	ARF17	Callose synthesis and pollen wall formation, anther development	Sun et al., 2015
	*Arabidopsis*	Down	ARF10/16/17	Somatic embryogenesis	Wojcik et al., 2017
miR847	*Arabidopsis*	Up	IAA28	Cell proliferation and lateral organ growth	Wang and Guo, 2015
miR319a	*Arabidopsis*	–	TCP4	Petal growth and development	Nag et al., 2009
miR398	Rice	Down	CSD1 and CSD2	Seed development, panicle length, grain number, and grain size	Zhang et al., 2017
miR397	Rice	Up	LAC	Grain size and panicle branching	Zhang Y. C. et al., 2013
miR397a	Pear	Up	LAC	Lignin synthesis, fruit development, and formation of fruit quality	Wu et al., 2014
miR396	*Arabidopsis*	Up	GRF/GIF	Pistil development	Liang et al., 2014; Jatan et al., 2020
	Rice	Up	GRF6	Semi-dwarf and leaf angle, plant architecture	Tang et al., 2018
miR172	Soybean	Up	AP2	Floral organ identity and flowering time, nodules number and nitrogenase activity	Yan et al., 2013
	Maize	–	AP2	adult phase and flowering	Chuck et al., 2007
	*Arabidopsis*	Up	AP2(TOE1, TOE2)	Developmental Timing, culm (main stem) and panicle development, fruit morphogenesis	Wu et al., 2009; Jose Ripoll et al., 2015
	Tomato	–	AP2a	Fruit ripening and development	Gao et al., 2015
miR165/166	*Arabidopsis*	–	HD-ZIP III	Vascular development and secondary cell walls formation	Du and Wang, 2015
miR164/169 (predicted)	Cucumber	–	NAC	Fruit-spine development	Liu et al., 2018
miR167a	Rice	Up	ARF6	Grain length and weight	Qiao et al., 2021
	Tomato	Up	ARF6 and ARF8	Floral development defects and female sterility	Liu et al., 2014

## miRNAs regulate plant architecture and yield

Due to the different spatial distribution of leaves, planting density has a significant effect on the photosynthetic efficiency of crops. The study of ideal plant architecture (IPA) is one of the key means to obtain elite high-yield crop varieties. Panicle branching and tillering are the two important determinants of plant architecture and high-yield potential in rice (Miao et al., 2019). IPA1 encodes miR156 target gene *SPL* (Secic et al., 2021), OsSPL14 is regulated by OsmiR156, and miR156 is a multifunctional miRNA; besides regulating plant architecture and yield in various plants, it also plays an important role in regulating the tillering, quality, and shape of rice grains (Jiao et al., 2010). Manipulation of miR398 also can increase panicle length, grain number, and size in rice, miR398 as a promising target for crop improvement (Zhang et al., 2017). Overexpression of MtmiR390 in *Medicago truncatula* resulted in promoting LR growth but preventing nodule organogenesis, rhizobial infection, and inhibiting the transcription of nodulation genes (Zhang et al., 2017). Overexpression of OsMIR396d in rice result in semi-dwarf and increased leaf angle, and BR signaling was enhanced. One of the OsmiR396d target gene OsGRF6 participated in interaction network of BR and GA signal transduction, controlling plant architecture (Tang et al., 2018). Similarly, overexpression miR397 improves grain growth and development, increases crop seed size, and promotes panicle branching by downregulating its target gene *LAC* in rice (Zhang Y. C. et al., 2013). Suppression of miR164 in maize led to an enhancement of auxin and gibberellin signaling, resulting in the elongation of internodes. TIR1 is a target of mi393 and is conserved among *Arabidopsis*, poplar, *Medicago*, and other plants. TIR1 binds Aux/indole-3-acetic acid (IAA) proteins in the E3 ubiquitin ligase SCF complex, which regulates multiple plant architectures (Bonnet et al., 2004).

Vegetative phase change is regulated by a decrease or increase in the abundance of the miRNAs in plants. Studies have verified that miR156 or miR157 specify the quantitative and qualitative changes in leaf morphology by regulating *SPL* gene during *Arabidopsis* vegetative phase (He et al., 2018). Other studies confirmed consistent results that the mature miR156 influences the number of leaves before the reproductive stage in the life cycle of plants (Kim et al., 2017). Furthermore, miR156-SPL modules play the pivotal roles in controlling shoot architecture and other traits in crops. Overexpressing GmmiR156b increased stem thickness significantly, as well as increased numbers of long branches, nodes, and pods, which exhibited an increased yield (Sun Z. et al., 2019). MiR156-targeted *SPL/SBP* transcription factors regulate *Arabidopsis* heteroblastic development and tomato ovary and fruit development (Ferreira e Silva et al., 2014; Nguyen et al., 2017). Overexpressing miR156 resulted in numerous positive traits, such as improved forage yield, delayed flowering, increased root length, and elevated biomass production in alfalfa (Aung et al., 2015). In addition, overexpression of miR156 caused a drastic phenotype, resulting in affected plant architecture and reduced tuber yield in potato. Also, root phenotypic changes are observed in *Medicago truncatula* and rice miR160 overexpression lines (Bustos-Sanmamed et al., 2013).

At the same time, miR156 overexpression in potato plants also exhibited altered levels of cytokinin and strigolactone, suggesting that miRNAs are likely to affect plant growth and development by regulating hormone signaling pathways (Bhogale et al., 2014). Besides, miR156 represses the floral development in *Arabidopsis* by positive regulators AGAMOUS-like proteins AGL15 and AGL18 double mutants, and it is worth mentioning that not in the single mutants, which indicated that AGL15 and AGL18 co-regulate miR156 expression (Serivichyaswat et al., 2015). miR156 regulates seed dormancy and grain yield by the gibberellin pathway in rice (Miao et al., 2019).

## miRNAs regulate organ development and flowering time of plants

Flowering is a key event in the plant life cycle and flowering time is regulated by multiple pathways. miRNA and its target genes form a complex regulatory network to participate in these pathways. Overexpression of miR172 caused accelerated vegetative development and resulted in an early flowering phenotype in *Arabidopsis* (Wu et al., 2009). It has been reported that *AP2* transcription factors are targeted by miR172, which involved in regulating flowing time, accelerating the floral organ development, and increasing the number of nodules in soybean. What is distinct is that miR172 is conductive to the leguminous crop nodulation and increases nitrogenase activity (Yan et al., 2013). Besides, *HD-ZIPIII* transcription factors as the target of miR165/166 played an important role in regulating plant vascular development and secondary cell wall formation (Du and Wang, 2015).

What is more, miR160 also plays a critical role in plant embryo development. MiR160 negatively regulating the auxin response factors *ARF10, ARF 16*, and *ARF 17* in *Dimocarpus longan*. Studies indicated that the pri-miR160 was downregulated in response to salicylic acid but upregulated by gibberellic acid, ethylene, and methyl jasmonate treatments, suggesting that pri-miR160 was associated with hormone signaling transduction (Lin et al., 2015). Overexpressed miR160 to silence a set of repressor auxin response factors resulted in cytokinin and auxin insensitivity, and inhibition of symbiotic nodule development in soybean (Turner et al., 2013). Importantly, miR160 targets the *ARF* transcription factors (*ARF10/ARF16/ARF17*) that control the auxin signaling pathway and plays a key role in somatic embryogenesis in *Arabidopsis* (Wojcik et al., 2017). Similarly, miR847 upregulates auxin signal pathway and then modulates cell proliferation and lateral organ growth in *Arabidopsis* (Wang and Guo, 2015). Not only that controlling leaf development, miR319a also regulates petal and stamen growth and development by regulating *TCP* transcription factors in *Arabidopsis* (Nag et al., 2009). Nevertheless, miR396 plays a critical role in promoting pistil development by the *GRF* or *GIF* transcription factors (Liang et al., 2014). In *Medicago truncatula*, miR396 negatively regulates the expression of six *MtGRFs* and two *bHLH79*-like genes implicate in the regulation of plant leaf growth (Wojcik et al., 2017).

## miRNAs regulate fruit and grain quality

Transcriptional repressor AP2 and its closest paralogue TOE3 as target genes of miR172 are critical for fruit growth, and ARF8 and ARF6 act upstream directly activating the expression of a miR172-encoding gene (MIR172C) to promote fruit valve growth. MADS/ARF–miR172–AP2 is a novel and most likely conserved miRNA-dependent regulatory module that links *Arabidopsis* fruit morphogenesis and hormonal pathways in the regulation of fruit growth (Jose Ripoll et al., 2015). Tomato fruit ripening and development are regulated by a key transcription factor ripening inhibitor (RIN), which binds directly to the promoter regions of MIR172a. RIN and miRNA expression as well as ethylene signal transduction co-regulate fruit development and ripening (Gao et al., 2015). Furthermore, OsARF6 is an upstream transcription factor of auxin response elements OsAUX3, and OsARF6 is targeted by the miR167a. Whereas, OsARF6 binds directly to OsAUX3 promoter, grain weight and length are regulated by a novel module miR167a-OsARF6-OsAUX3 in rice (Qiao et al., 2021).

Fruit spines are an important trait for fruit quality in cucumber (*Cucumis sativus* L.). *NAC* transcription factors were predicted to be the targets of several miRNAs (miR164 or miR169) by GO enrichment analysis. These findings indicated that miRNAs have an impact on fruit-spine development by being involved in regulating the expression of *CsvNAC* genes (Liu et al., 2018). Over expression of Ptr-miR397a and 17 *PtrLACs* was downregulated, resulting in lignin content reduced in pear. KEGG pathway analysis showed that miRNAs are involved in pear fruit development and formation of fruit quality by regulating pear fruit lignin synthesis, sugar, and acid metabolism, as well as hormone signaling (Wu et al., 2014). Therefore, miRNAs can improve agronomic traits by regulating secondary metabolic pathways in plants.

### miRNAs regulate plant fertility

Male sterility is a very useful agronomic trait for heterosis utilization and hybrid seed production (Wan et al., 2019). In hybridization breeding, high-yield hybrid varieties can be obtained using male sterile lines to control pollination. The disorder of anther cell differentiation can lead to pollen abortion and male sterility. miRNAs that regulate plant fertility are mostly related to pollen and flower development (Sun et al., 2015). Therefore, miRNA-based strategies provide a new opportunity for molecular breeding for high-yield crops.

AtTTP is involved in miR160 maturation in *Arabidopsis* anther, and both of them co-regulate downstream target gene ARF17expresssion and promote callose synthesis and pollen wall formation. Thus, overexpression AtTTP caused thinner callose wall and reduced male fertility (Sun et al., 2015). The interesting thing is that in addition to male sterility, miRNAs are also involved in female sterility. Overexpressing *Arabidopsis* miR167a shortened stamens, petals, and styles in transgenic tomato plants, decreased expression of target genes ARF6A and ARF8A/B, led to floral development defects, and resulted in female sterility (Liu et al., 2014). Earlier studies have shown that miR172 has been demonstrated to promote adult phase and flowering, whereas miR156 is involved in juvenile stage development in maize (Chuck et al., 2007).

For monoclinous and autogamous crops, such as rice and wheat, artificial and mechanical emasculation of female parent plants is inefficient, and the three-line hybrid system (male sterile lines, restorer lines, and maintainer lines) has higher requirements for germplasm resources (Wang and Deng, 2018). Thus, to address limited germplasm issue and simplify the tedious hybrid seed production process, the previous study provides a valuable resource of photoperiod- and thermo-sensitive genic male sterile (PTGMS) rice line fertility-related genes, though PTGMS system is affected by the environment (Song et al., 2021). The role of miRNA-mediated fertility transition and PTGMS development are in demand. Consequently, the research revealed that miR156a, miR5488, and miR399d have prominent contributions in the regulation of fertility changes by involving in the development of anthers and male sterility in PTGMS rice (Sun et al., 2021). The loss of miR2118 exhibited abnormal development in somatic anther wall cells, which caused severe male and female sterility in rice (Araki et al., 2020). It is reported that new microRNAs tae-miR2275-CAF1 and tae-miR1127a-SMARCA3L3 are involved in regulating anther development in male sterile wheat (Sun et al., 2018).

In short, miRNA is a promising target for the genetic improvement of crop architecture and yields, as well as quality. As the effective regulatory strategy, miRNA directly and indirectly controls growth and development in plants, profoundly improves agronomic traits, and substantially enhances grain yield. More profound studies provide a significant step forward in understanding the miRNA-mediated regulation mechanism and increasing the available genetic diversity in breeding germplasm (Tester and Langridge, 2010).

### Roles of miRNA encoded peptide in plants

For a long time, miRNAs are considered the class of non-coding small RNAs. However, recent studies have shown that primary miRNAs (pri-miRNAs) can encode for regulatory peptides, called miRNA-encoded peptides (miPEPs), which specifically enhance the levels of mature miRNAs by increasing the transcription of corresponding pri-miRNA (Lauressergues et al., 2022). So far, miPEPs have been reported in plant species and even in animals (Dozier and Plaza, 2022). The miPEPs are proved to be a powerful agronomic tool without dealing with genomic manipulations (Yadav et al., 2021a). miRNAs being associated with miPEPs could be an interesting study in different crop plants for agronomic traits improvement, such as primary root length and nodulation in legumes (Jatan and Lata, 2019). Since miPEPs are very specific for each miRNA, the specificity of miPEPs may potentially represent an advantage in regulating the plant growth and development.

External application of synthetic miPEPs by spraying, watering, composting, and adding fertilizer increases the miRNA expression and downregulates the target gene (Yadav et al., 2021b). Exogenous application of synthetic miPEP172c (0.1 μM, watering plant three times a week during nodulation) led to miR172c expression, thereby downregulating its target gene, the AP2 transcription factor NNC1 more actively. These data suggest that miPEP172c could stimulate nodulation in soybean (Couzigou et al., 2016). Similarly, the exogenous application of miPEP164c to suspension-cultured grape berry cells enhanced the transcription of its corresponding non-mature miR164c, with a maximum effect at 1μm and after a period of 10 days, thus leading to a more obvious repressing of its target gene, the grapevine transcription factor VvMYBPA1. This led to a significant inhibition of the proanthocyanidin biosynthetic pathway while simultaneously stimulating anthocyanin biosynthetic. Anthocyanin content was 31% higher in miPEP164c-treated cells (Vale et al., 2021). Another example of miPEPs affecting plant secondary metabolism is that miPEP858a controls flavonoid biosynthesis in *Arabidopsis*. The mutation of the miPEP858a coding region caused a decreased expression of miR858a, and miPEP858a-edited plants were obtained. The exogenous treatment of the miPEP858a-edited plants with synthetic miPEP858a (0.1 to 0.25 μM) complemented the phenotypes and the gene function (Sharma et al., 2020). These studies suggest the importance of miPEPs in exerting control over plant growth and development. It has been proven that vvi-miR171d acts as a key regulator in the formation of adventitious roots in grapevine. After exogenous treatment with miPEP171d1, the transcription of vvi-MIR171d and the number of adventitious roots were increased in the grape tissue culture plantlets. However, exogenous application and overexpression of MtmiPEP171d1 did not result in phenotypic changes in *Arabidopsis*. On the contrary, ath-miPEP171c, encoded by the *Arabidopsis* ortholog of vvi-MIR171d, induced the adventitious roots in *Arabidopsis*, while it had no effect on grape root development (Chen Q. J. et al., 2020). These results further suggest the species specificity of miPEPs.

## Conclusion and perspectives

It is of great significance to study the function of miRNAs, especially in plant growth and development, for the breeding of crops varieties with high quality, high yield, and stress tolerance. More and more miRNAs and their target genes have been predicted and verified by bioinformatics and molecular biology approaches in plants. miRNAs regulate at post-transcriptional level, particularly transcription factor combined directly with conservative cis-regulatory promoter seems to be more general. However, since most of these target genes are transcription factors, the mechanism of miRNA involved in plant stress response is more complex. Based on this, plant miRNAs have emerged as the promising targets for crop improvement, because they can control intricate agronomic traits, which give a positive regulation for better yield, quality, and stress tolerance (Zhang et al., 2017). What elucidated above may contribute to prove the importance of miRNAs for plants. Transcription factors as one of the target genes of miRNAs have multiple transcriptional activation functions according to their subunits, which have paramount importance in regulating plant progress and acclimation, and another target is gene encoding proteins or enzymes involved in plant metabolic regulation (Li, 2015; Wang et al., 2016; Samad et al., 2017). Overexpression or repression of the miRNAs could lead to downregulation or upregulation of their downstream target genes, consequently resulting in pleiotropic phenotypes in plants. To obtain a proper understanding, synchronously carried out studies on miRNAs and their target mRNAs are needed.

Degradome sequencing technology can be used by prediction and evaluation of miRNA candidates, which is useful understanding the post-transcriptional role of miRNAs. The use of artificial miRNAs (amiRNAs), effective strategies for silencing endogenous genes, will be the efficient method to specifically inhibit the expression of target genes for determining the functions of miRNAs in both developmental regulation and response to abiotic stresses. Furthermore, it is reported that amiRNAs-mediated gene silencing has helped in explaining functional importance in many genes. This technology also has been applied for the improvement of agronomy performance in various plants. Different expression strategies are closely related to various phenotypes in transgenic plants (Yu et al., 2019; Singroha et al., 2021). Short tandem target mimic (STTM) technology is an another highly effective and specific strategy for induced degradation of multiple miRNAs, which can be used as a powerful tool for functional dissection of miRNAs research, and strongly promotes the study of miRNA regulatory network functions and interactions (Zhang et al., 2017; Yang et al., 2019). STTM lines are the valuable resource and potential breeding materials for functional miRNA studies (Zhang et al., 2017). Furthermore, gene-editing techniques can knock out or replace specific non-coding miRNAs, miRNAs and their targets may be combined with gene-editing technology to improve agronomic traits, which provide possibility in cultivating various species, and transgenic plants can be obtained. The emergence of genome-editing technology will obtain a large number of mutants and enrich experimental resources. CRISPER/Cas9 has been proved to be an effective and important method for editing miRNA in plants, which will greatly expand the application of miRNA in crop molecular breeding (Zhou et al., 2017). According to the above, there are some biotechnological tools to modulate miRNA expression, for example, regulating artificial miRNA genes and endogenous miRNAs, CRISPR/Cas-mediated miRNA gene editing, and miRNA-based SSR markers for improving stress tolerance (Shriram et al., 2016; Zhu, 2016).

The target genes of all differentially expressed miRNAs can be predicted using psRNA target with a plant transcriptome dataset and identified through experimental techniques. Through target prediction, downstream targets of miRNAs can be identified, revealing a novel function for miRNA in plants. However, compared to conserved miRNAs, many less-conserved plant miRNAs have predicted targets that have proven difficult to confirm (Meyers et al., 2008). The roles of novel and conserved miRNAs that are being revealed and their predicted functions should be considered.

The phenomena of a combination of two or more different stresses on plants, such as the combination of drought and high temperature, are general. miRNA regulation is a potential strategy for improving tolerance to abiotic stress combination in crop plants (Zandalinas et al., 2018). Another interesting thing is that downregulated expression of miR159 was observed under high temperature in wheat, but presented an opposite regulatory trend in switchgrass (Zhu et al., 2019). The same miRNA family members demonstrate different expression patterns. Diversity of expression pattern could contribute to the species-specific miRNA regulation, or that miRNAs respond to abiotic stress only in specific tissues or at specific developmental stages. Furthermore, miRNA-mediated regulatory mechanism underlies epigenetic memory formation in plants. Interestingly, on the one hand, miRNA as epigenetic modulators, targeting epigenetics-associated enzymes, affects the protein level of target genes without changing gene sequences. On the other hand, the expression of miRNA could be regulated by epigenetic modifications, including DNA methylation, RNA modification, and histone modification (Gruber and Zavolan, 2013; Yao et al., 2019). Recent research indicated that epigenetic mechanisms are intricately linked with stress memories and adaptation in plants (Kinoshita and Seki, 2014).

A preliminary statistical analysis shows that several miRNAs (miR156, miR159, miR160, miR164, and miR172) are most studied miRNA families that showed diverse roles in diverse stresses and diverse plant species (Table 1). miR156-targeted SPL TFs modulate plant architecture, including grain size, panicle branching, and higher grain productivity in rice (Jiao et al., 2010; Miura et al., 2010; Wang S. et al., 2012), and modulate plant architecture and tuberization in potato and temporal regulation of shoot development in *Arabidopsis thaliana* (Gruber and Zavolan, 2013; Yao et al., 2019). The miRNA156-targeted SPL/SBP box TFs regulate tomato ovary and fruit development (Ferreira e Silva et al., 2014). The miRNA159-regulated MYB TFs in the regulation of programmed cell death in *Arabidopsis* (Alonso-Peral et al., 2010), floral development and stem elongation in rice (Tsuji et al., 2006), anther development and heat response in wheat (Wang Y. et al., 2012), leaf and floral development in tomato (Buxdorf et al., 2010), and targeting of isotrichodermin C-15 hydroxylase and its involvement in immune response of cotton (Zhang et al., 2016). The miR160 regulates a group of repressor ARFs, which are mainly involved in auxin hypersensitivity and regulation of floral organ development, seed germination, and post-germination stages in *Arabidopsis thaliana* (Liu et al., 2007, 2010), ovary patterning, floral organ abscission and lamina outgrowth in tomato (Turner et al., 2013), growth and developmental defects of rice (Huang et al., 2016), and inhibition of symbiotic nodule development in soybean (Turner et al., 2013). The miR164-directed cleavage of NAC1 mRNA affects lateral root development in *Arabidopsis* (Guo et al., 2005), maize (Li et al., 2012), drought resistance in rice (Fang et al., 2014), and boundary specification in tomato (Berger et al., 2009) and negatively regulates the resistance of wheat to stripe rust (Turner et al., 2013). miR172 suppressing AP2 genes induce flowering, spikelet determinacy, and floral organ abnormalities in rice (Zhu et al., 2009; Lee et al., 2014), promote vegetative phase change in maize (Lauter et al., 2005), regulate soybean nodulation (Yan et al., 2013), and affect cleistogamous flowering in barley (Nair et al., 2010) and graft-transmissible induction of potato tuberization (Martin et al., 2009). In the recent years, it has been suggested that the role of plant growth promotes rhizobacteria in modulating stress-responsive miRNA. In chickpea, miRNA-mediated drought and salt tolerance have also been reported under the influence of *Pseudomonas putida*. The expression patterns of miR159, miR160, miR166, miR396, miR393, etc. have significant alterations in drought and salt stress, which suggests that these miRNAs play a vital role in abiotic stress alleviation in chickpea (Jatan et al., 2019a,b). Moreover, miR164a-CUC1, miR167-NRAMP1, miR393a-5p-TIR1, and miR396a-5p-GRF1 modules might be involved in the regulation of root, leaf, and flower development of *Arabidopsis* during *Pseudomonas putida*–inoculation (Jatan et al., 2020). All of the above clearly indicate that those most studied miRNA families play a role at the core of gene regulatory networks in regulating the growth and development and stress response of plants.

Despite miRNA-based regulatory mechanisms and biological functions of target gene are yet to be investigated, we are still not making full and effective use of miRNA-based methods to improve plant protection strategies. Many challenges related to adversity on plant miRNA research still remain. A large proportion of conservative miRNA may involve in plant growth and development, and non-conservative (specific) miRNA participates in resisting abiotic stress. However, most of miRNAs reported are highly conserved in plants. Previous studies have showed that miRNAs exhibit temporal and spatial specificities in regulating gene expression and at the same time show specificity in different species, which play an important regulatory role in the growth and development of plant roots, stems, leaves, flower organs, and fruits, as well as in response to biotic and abiotic stresses (Tiwari et al., 2014; Zhao et al., 2021). At present, studies on miRNAs in plants are mainly focused on model plants *Arabidopsis* or rice, whereas studies on other important crops are still relatively scarce, especially species-specific miRNAs that have a lot of room for further discovery. In addition, there are still a large number of miRNAs and their target genes related to agronomic traits that have not been discovered, and individual miRNA may play multiple roles in various abiotic stress and growing development regulatory pathways. Therefore, it is necessary to explore new miRNAs, reveal their target genes, and further analyze the miRNA-mediated regulatory network. miRNA-based strategies are used to improve signal transduction pathway and metabolism network regulation, so as to stimulate adaptive behavior in plants. How do plants cope with the harsh environment? “Combat with” or “adapt to”? Decipher this mazy mechanism of miRNA-dependent stress tolerance is promising in plants. It provides technical support for genetic improvement of crops and promotes sustainable development of agricultural production.

## Author contributions

FZ, JY, and HS drafted, wrote, and edited this review. JW and NZ participated in the discussion of this review. All authors have read and agreed to the published version of the manuscript.

## Funding

This research was funded by the National Natural Science Foundation of China (Grant Nos. 31860400 and 31960444) and State Key Laboratory of Aridland Crop Science, Gansu Agricultural University (Grant No. GSCS-2017-6).

## Conflict of interest

The authors declare that the research was conducted in the absence of any commercial or financial relationships that could be construed as a potential conflict of interest.

## Publisher's note

All claims expressed in this article are solely those of the authors and do not necessarily represent those of their affiliated organizations, or those of the publisher, the editors and the reviewers. Any product that may be evaluated in this article, or claim that may be made by its manufacturer, is not guaranteed or endorsed by the publisher.
